# Effect of antioxidant-rich kindergarten meals on oxidative stress biomarkers in healthy 5–6-year-old children: a randomized controlled trial

**DOI:** 10.1007/s00431-024-05576-6

**Published:** 2024-04-25

**Authors:** Maja Berlic, Mojca Korošec, Žiga Iztok Remec, Vanja Čuk, Tadej Battelino, Barbka Repič Lampret

**Affiliations:** 1 Department of Food Science and Technology, Biotechnical Faculty, University of Ljubljana, Ljubljana, Slovenia; 2 Preschool Galjevica, Ljubljana, Slovenia; 3 Clinical Institute for Special Laboratory Diagnostics, University Children’s Hospital, University Medical Centre Ljubljana, Ljubljana, Slovenia; 4 Department of Endocrinology, Diabetes and Metabolic Diseases, University Children’s Hospital, University Medical Centre Ljubljana, Ljubljana, Slovenia; 5 Faculty of Medicine, University of Ljubljana, Ljubljana, Slovenia

**Keywords:** Antioxidant-rich diet, Oxidative stress biomarkers, Dietary antioxidant capacity, Reactive oxygen metabolites, F2-isoprostanes, Kindergarten diet

## Abstract

As children spend up to 9 h a day in kindergarten, the main purpose of our study was to evaluate the effect of antioxidant-rich kindergarten meals on oxidative stress biomarkers (OSBs) in healthy children. In the randomized control trial with a follow-up, healthy 5–6-year-old children from six kindergartens were randomly divided into a prototype group (PG, *n* = 40) and a control group (CG, *n* = 17). PG followed a 2-week antioxidant-rich kindergarten meal plan (breakfast, lunch, and two snacks), and CG followed their standard kindergarten meal plans. Outside the kindergartens, participants ate as usual. We used a consecutive 7-day dietary record inside and outside the kindergarten and the national dietary assessment tool OPEN to assess the total dietary antioxidant capacity (dTAC) of the consumed foods. Malondialdehyde (MDA), 8-hydroxy-2-deoxyguanosine (8-OHdG), and four F2-isoprostane were measured in fasting urine on days 1 and 15. We also measured total antioxidant power (PAT) and hydroperoxides (d-ROMs) in fasting serum on day 15 and obtained the value of the oxidative stress index (OSI). We used a Welch two-sample *t*-test and multiple regression analysis to compare the prototype and control groups and a nonparametric Wilcoxon signed rank exact test to compare pre- and post-intervention results in urine. Antioxidant-rich kindergarten meals contributed to a significantly (*p* < 0.05) higher intake of dTAC in PG participants compared to standard meals in CG participants (8.6 vs. 2.8 mmol/day). We detected a negative correlation between dTAC intake and d-ROMs and between dTAC intake and OSI (*r* =  − 0.29, *p* = 0.043 and *r* =  − 0.31, *p* = 0.032, respectively). A significant decrease in urinary 8-iso-15-prostaglandin-F-2 alpha was detected in PG participants between days 1 and 15; however, no other intra-individual significant differences in urinary OSBs were found.

*  Conclusion*: Antioxidant-rich food in kindergarten is warranted due to its potential health-protective effect. Additionally, we present original data on the average levels of urinary and serum OSBs in healthy 5–6-year-old children.

*  Trial registration*: The study was registered at ClinicalTrials.gov, on February 5, 2020 (https://clinicaltrials.gov/ct2/show/NCT04252105).

**Table:** 

**What is Known:** *• Kindergartens are recognized as promising environments for public health measures.* *• A diet rich in antioxidants can reduce OSBs and, consequently, the risk of developing NCDs.*	
**What is New:** *• Antioxidant-rich kindergarten diet can ensure a protective intake of dTAC in children.* *• Original data on serum oxidative stress biomarkers (d-ROMs, PAT, and OSI) and urinary oxidative stress biomarkers (MDA, 8-OHdG, and F2 isoprostanes) in healthy 5–6-year-old children.*

**Supplementary Information:**

The online version contains supplementary material available at 10.1007/s00431-024-05576-6.

## Introduction

In recent years, obesity among preschool children has been on the rise [1, 2], and there is evidence that childhood obesity increases the risk of cardiovascular disease (CVD) in adulthood [3]. Noncommunicable diseases (NCDs) such as obesity, CVD, type 2 diabetes, and different types of cancer are the leading causes of death worldwide [4] and are closely related to oxidative stress [5].

Oxidative stress can be defined as an imbalance between the pro-oxidant and antioxidant species in favor of the former. An excessive increase in reactive oxygen species (ROS) on the one hand and an insufficient antioxidant defense on the other disrupt the redox balance, leading to extensive damage to cell membranes lipids, proteins, and DNA [6]. Oxidative injuries are closely aligned with the degradation of biomolecules, and their degradation products represent well-recognized markers of oxidative stress such as lipid oxidation products (malondialdehyde (MDA) and F2-isoprostanes), DNA/RNA oxidation products such as 8-oxo-2′-deoxyguanosine, and protein oxidation products such us 3-nitro-tyrosine [7]. While previous methods for determining oxidative stress biomarkers (OSBs) in urine focused on a single marker, a highly accurate and precise method for simultaneous determination of seven biomarkers of oxidative damage of lipids, proteins, and DNA has recently been presented and enables a better insight into the oxidative state of an organism [8]. In addition, evaluation of the oxidative stress level can be performed by measuring the plasma/serum levels of derivatives of reactive oxygen metabolites (d-ROMs) and of the biological antioxidant potential (BAP/PAT) using the same sample and testing equipment [9]. The information derived from the d-ROM and the PAT analysis can be summarized into one single value called oxidative stress index (OSI) [10].

However, epidemiologic evidence indicates that the consumption of antioxidant-rich foods, such as vegetables, fruits, whole grains, legumes, nuts, and spices [11], may reduce the risk of all-cause mortality [12, 13]. In addition, it is associated with many beneficial effects on children’s health related to maintaining telomere length [14], obesity [15, 16], allergic diseases [17], and lung function [18]. Consumption of dietary antioxidants can augment cellular redox balance [6] as they activate antioxidant signaling, improve endogenous antioxidant status, and suppress oxidative stress [19]. To assess the antioxidant capacity of the whole diet, the concept of dietary total antioxidant capacity (dTAC) was introduced, which is also a suitable indicator of dietary antioxidant properties [20].

In high-income countries, 83% of all children are enrolled in pre-primary education [21]. In Europe, more than 90% of 5–6-year-old children stay up to 9 h a day in kindergarten, where they consume up to 75% of their daily energy intake and nutritional needs [22]. Due to a large number of enrolled children, in recent years, preschool and school environments have been recognized as promising environments to promote healthy eating habits among children [23, 24]. Namely, many observational studies have shown that positive changes in health behavior can be made in early childhood [23], contributing to better health later in life [24, 25]. 

To the best of our knowledge, no studies have assessed the contribution of daily kindergarten meals on the average dTAC intake in healthy children and their effect on OSBs. Therefore, the primary aim of our study was to examine the potential link between antioxidant-enriched kindergarten meals and urinary and serum OSBs in healthy 5–6-year-old children. We hypothesized that antioxidant-rich kindergarten meals can reduce urinary OSBs.

## Methods

### Study design

Our 2-week randomized control trial with a follow-up included participants from six kindergartens situated within urban and suburban areas of Central Slovenia. A written consent was obtained from the kindergarten management teams prior to participation in the study. Kindergartens were randomized into two groups, prototype group (PG) and control group (CG). Detailed information regarding the randomization procedure is described in our previous study [26]. Setting the targeted statistical power of the analysis at 90% with a two-sided test alpha (α) value of 0.05 and assuming a dropout rate of 10% and a sample size ratio between CG and PG of 0.5, the required number of participants per group to detect a difference of 5000 µmol TE/dan in daily dTAC consumption in kindergarten was 23 in PG and 12 in CG. The study was scheduled to take place between the end of February 2020 and the end of April 2020, but due to the COVID-19 epidemic, we conducted it between May and June 2020.

The study was registered at ClinicalTrials.gov (NCT04252105). Ethical approval was obtained from the Committee for Medical Ethics of the Republic of Slovenia (No. 0120–66/2019/8).

### Intervention dietary design

Nutrition in Slovenian kindergartens is systematically regulated, and children do not bring food from home as, according to the Guidelines for Healthy Eating in Educational Institutions [27], all meals are prepared within the kindergarten facilities. Before the intervention started, a 10-day antioxidant-rich meal plan was designed (prototype meal) in which staple food with high dTAC was included [11] and sent to PG kindergartens (Supplementary Table S1). Kindergartens in the CG used the meal plan from the same period of the previous year to avoid bias due to awareness of their participation in the study (Supplementary Tables S2-S3). Parents were explicitly instructed not to alter their household diet during the study intervention.

### Study population

Written parental consent was obtained before the study started. Parents and children did not receive any financial compensation for their participation. The eligibility criteria were as follows: a healthy child, aged 5–6 years, attendance at all kindergarten meals (breakfast, lunch, and two snacks) during the intervention period, provision of biological samples (urine samples were mandatory, and blood samples were highly preferred), and a fully recorded consecutive 7-day dietary record. Gender was not important. To prevent an insufficient number of final participants resulting from potential dropouts during the research, we initially invited all children from two classrooms in each kindergarten. The exclusion criteria were all food allergies, chronic disease, and the use of dietary supplements.

### Anthropometric measurements

Data on body mass and height were obtained from children’s pediatricians during routine medical examination prior to school enrolment, planned for all participants on day 15 of the intervention. The BMI was calculated as weight (kg)/height^2^(m^2^), and the weight status was defined through the International Obesity Task Force—sex and age specific cut-offs for children [28].

### Dietary assessment and dTAC calculation

A 7-day consecutive dietary record in and out of kindergarten was obtained from each participant. The detailed methodology is described in our previous study [26]. Dietary records were checked within 1 week after intervention for errors or missing details. If any were found, a dietitian contacted parents or teachers by phone to gather the needed information. dTAC of consumed food was calculated using Slovenian dietary assessment tool OPEN (http://opkp.si/), where more than 90% of foods contain data on dTAC, calculated based on the oxygen radical absorbance capacity (ORAC) of each food reported by the US Department of Agriculture and expressed in micromoles trolox equivalent (µmol TE)/100 g of food [29]. To assign a TAC value for foods not available in OPEN, the data for a similar food item in the same botanical group were used as a proxy.

### Biological sample collection

Fasting blood samples on day 15 of the intervention were collected at the participant’s nearest authorized laboratory of the health center, centrifuged for 10 min at 3500 g, and stored separated plasma at − 20 °C. The number of blood samples (*n* = 49) and urine samples (*n* = 114) in the study differed since urine was sampled twice from all participants, while eight participants refused the single collection of blood. Fasting urine samples on days 1 and 15 were taken by participants at home, in 50 mL polypropylene containers. They were delivered to the nearest authorized laboratory of the health center, centrifuged for 5 min at 3500 g, and stored at − 20 °C immediately (maximum 2 h after collection). The frozen samples were transferred to the special laboratory of the University Children’s Hospital in Ljubljana, Slovenia, on the same day and frozen at − 80 °C until analysis. Measurements were performed in samples thawed only once.

### Serum assays

Total cholesterol, HDL and LDL cholesterol, and high sensitive C-reactive protein level (hs-CRP), as data on lifestyle-related factors, were measured using an automatic analyzer (Siemens ADVIA 1800 Chemistry System, Germany). All analyses were performed within 5 months of collection. Uric acid, creatinine, and total bilirubin were measured using standard procedure (results obtained from Synlab, Ljubljana, Slovenia).

Oxidative activity was assessed by measuring levels of d-ROMs and antioxidant activity by measuring PAT using automatic FRAS5 photometric analytical device (H&D srl, 43,124 Parma, Italy [30]) with a colorimetric diagnostic kit (KIT d-ROMs FAST Test and KIT PAT Test), following the manufacturer’s instructions [31]. Subsequently, FRAS5 summarized the results obtained from the d-ROMs and the PAT test to provide the OSI value. The summary of the method is described in the Supplementary material (Methods for determining serum OSBs).

### Urinary assay

Simultaneous determination of six OSBs in urine was performed following the method described by Martinez and Kannan [8], with some modifications. The summary of the method is described in the Supplementary material (Method for determining urinary OSBs, Table S4 and Table S5).

### Statistical analysis

All data were expressed as mean with standard deviation (SD), minimum and maximum, and median and interquartile range (IQR), when data were not normally distributed. All analyses were two-sided, with statistical significance set at alpha = 0.05. Normal distribution of data was checked by visual inspection of boxplots and quantile–quantile (QQ) plots. All data were approximately normally distributed. We performed a Welch two-sample *t*-test to compare differences between data obtained in participants from the PG and the CG in the cases where data were normally distributed. We performed multiple linear regression analysis for serum OSBs and included gender as a confounder. We checked the distribution of the residuals before the analysis, and we used logarithmic transformation of OSI and PAT variables to increase the fit of the model. Associations of measurements among individual pairs of urinary variables were tested with the nonparametric Wilcoxon signed rank exact test. To test the strength of correlation between two variables, we used the Pearson product-moment correlation coefficient. All analyses were performed using R Statistical Software (v4.2.2; R Core Team 2022) [32].

## Results

### Study population

**Fig. 1:**
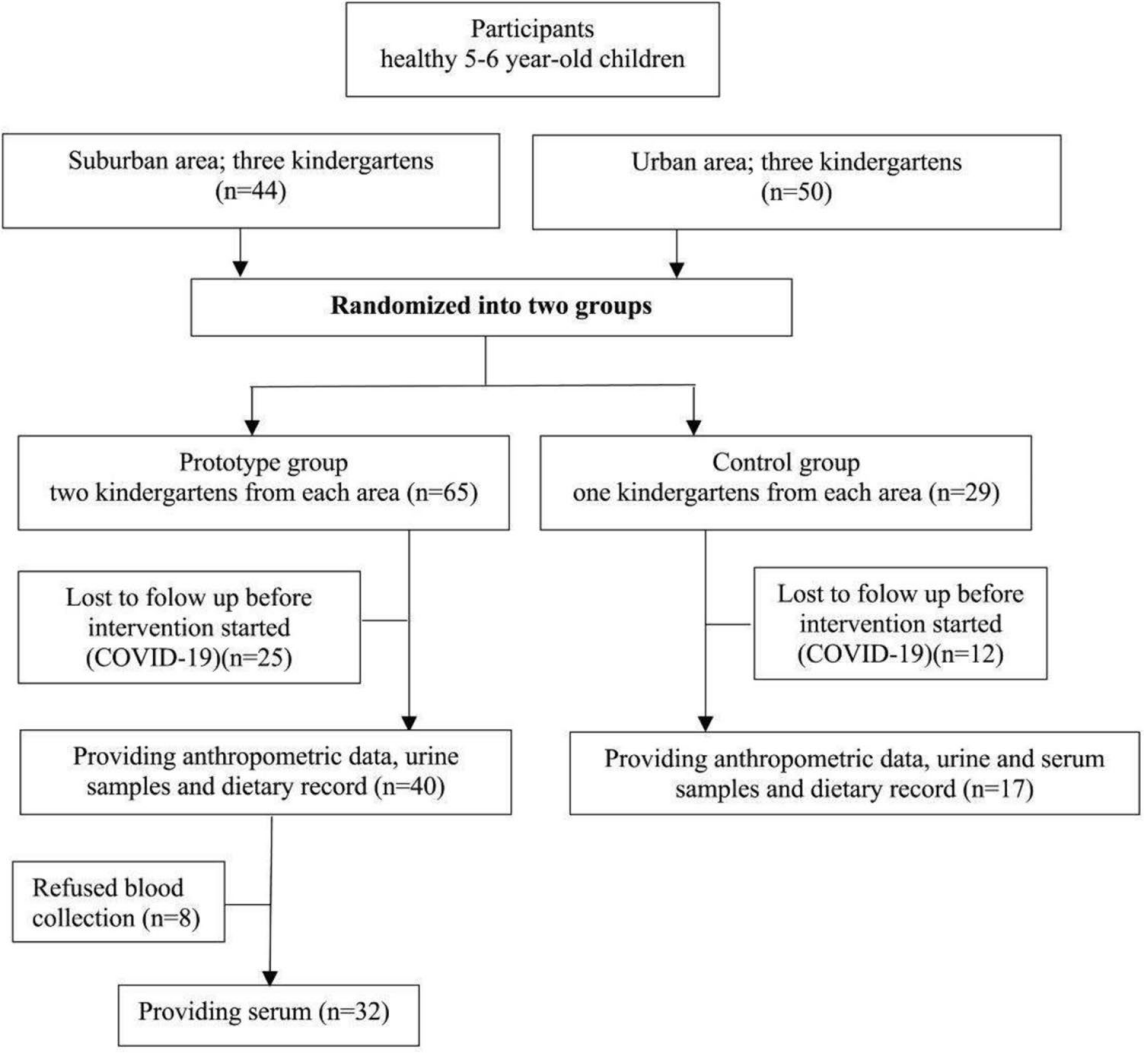
Participant’s flowchart

From the initial 94 obtained approvals, 57 (36 boys and 21 girls) healthy 5–6-year-old Caucasian participants successfully completed the 15-day intervention trial (61% response rate). Of these, eight participants refused blood collection due to the COVID-19 epidemic (all from the PG) but remained part of the study population by providing anthropometrics data, urine samples, and dietary records (Fig. 1).

### Anthropometric and serum measurements

In PG and CG participants, we measured similar basic anthropometric and basic serum characteristics with the exception of uric acid (Table 1).

**Table 1:** Anthropometric details and serum measurements in healthy 5–6-year-old participants

	Prototype group n=40^a^,n=32^b^ mean ± SD^c^	Control group n = 17^a,b^ mean ± SD^c^	p value
Gender (boys/girls (%))	17/15 (47)	13/4 (24)	
Height (m)^a^	1.18 ± 0.05	1.18 ± 0.03	*p *= 0.650
Body mass (kg)^a^	22.23 ± 3.57	21.54 ± 3.69	*p* = 0.212
BMI (kg/m^2^)^a^	15.94 ± 1.76	16.40 ± 2.60	*p* = 0.910
IOTF (percentile)	58.3 ± 27.55	46.8 ± 24.79	*p* = 0.142
Total cholesterol (mmol/L)^b^	4.12 ± 0.57	4.16 ± 0.74	*p* = 0.822
LDL (mmol/L)^b^	2.33 ± 0.47	2.32 ± 0.61	*p* = 0.947
HDL (mmol/L)^b^	1.49 ± 0.32	1.58 ± 0.21	*p* = 0.275
hs-CRP (mg/L)^b^	0.34 ± 0.48	0.36 ± 0.47	*p* = 0.874
Creatinine (µmol/L)^b^	41.5 ± 2.32	41.05 ± 2.9	*p* = 0.564
Uric acid (µmol/L)^b^	214 ± 34	240 ± 39	***p* = 0.022**
Bilirubin (µmol/L)^b^	6.5 ± 3.1	7.4 ± 2.6	*p* = 0.302

Values in bold indicate significant difference

*BMI*, body mass index; *IOTF*, international obesity task force; *hs-CRP*, highly sensitive C-reactive protein

^a^anthropometric measurements

^b^serum measurements

^c^data are expressed as mean ± standard deviation

### Dietary antioxidant intake

**Fig. 2:**
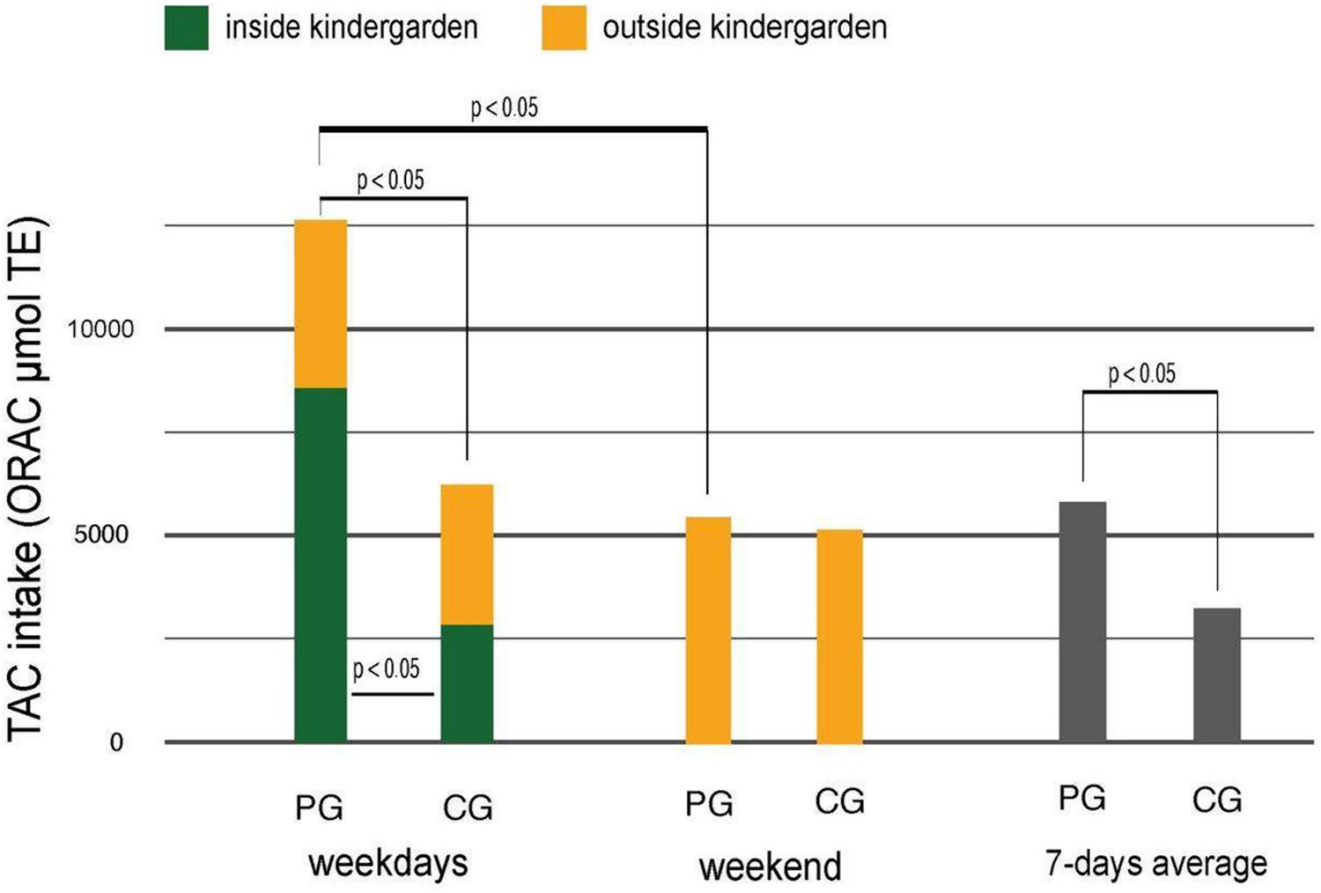
Comparison of total antioxidant capacity (TAC) intake among study groups based on their eating places

With kindergarten meals, PG participants consumed significantly (*p* < 0.05) higher dTAC than CG participants (8558 vs. 2831 µmol TE), while dTAC of the food consumed outside the kindergarten was similar for participants in both study groups. During the weekend, PG participants consumed significantly (*p* < 0.05) lower levels of dTAC compared to weekdays, whereas these differences were not statistically significant for CG participants. Due to the high dTAC intake in kindergarten, PG participants also consumed significantly higher average 7-day dTAC compared to CG participants (Fig. 2). Data in Supplementary Table S6 show that PG participants consumed 74% of their mean weekday dTAC intake (11.612 µmol TE) with kindergarten meals, whereas CG participants consumed a mean of 5.499 µmol TE during weekdays, of which approximately 51% was consumed in kindergarten.

### Serum OSB levels

**Fig. 3:**
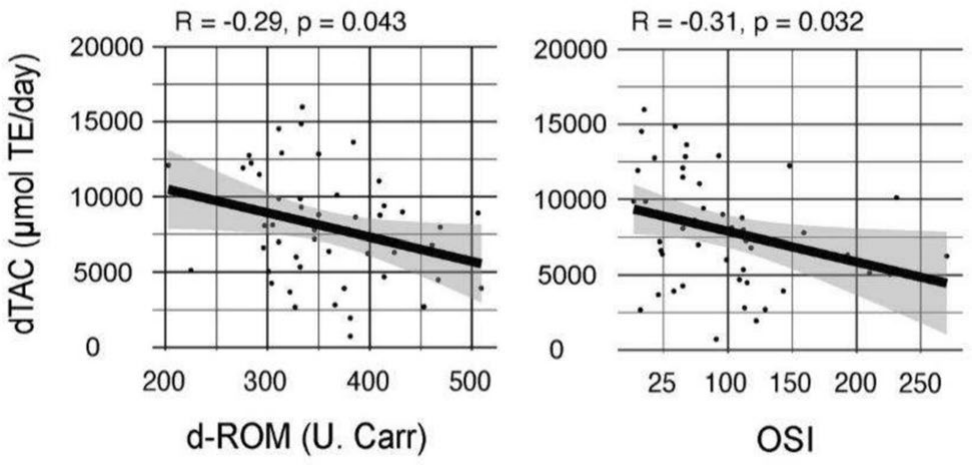
Correlation between dTAC intake and serum OSBs. dTAC, dietary total antioxidant capacity of all consumed food; d-ROM, serum oxidative stress biomarkers; d-ROM test-unit of measure-U.Carr = 0.08 mg H_2_O_2_/dL; OSI, oxidative stress index

We observed no statistically significant difference between participants in the PG and CG groups regarding the values of serum OSBs (Table 2), when controlling for gender as a confounder (Table 3). However, a weak yet significant negative correlation emerged between dTAC intake and d-ROM, as well as between dTAC intake and OSI (*r* =  − 0.29, *p* = 0.043 and *r* =  − 0.31, *p* = 0.032, respectively) (Fig. 3).

**Table 2:** Serum oxidative stress biomarkers in healthy 5–6-year-old children

	Prototype group n = 32 mean ± SD^a^	Control group n = 17 mean ± SD^a^
d-ROMs (U.Carr.)	345 ± 65	380 ± 66
PAT (U.Cor)	3510 ± 904	3543 ± 780
OSI	89 ± 63	106 ± 45

*dROMs*, reactive oxygen metabolites; *PAT*, total antioxidant power; *OSI*, oxidative stress index

^a^data are expressed as mean ± standard deviation

**Table 3:** Multiple linear regression results: effect of kindergarten meals on serum OSBs when controlled for gender

Regression model		Estimate	Std Error	*t* value	*P*(sig)
log(OSI) ~ kindergarten meals + gender	Intercept	4.358	0.1367	31.891	<0.001
	Kindergarten meals (CG)	0.332	0.178	1.866	0.069
	Gender (male)	-0.146	0.174	-0.840	0.405
Adjusted *R*-squared 0.034, *F*-statistic 1.821 on 2 and 46 DF, *p* value 0.174					
log(PAT) ~ kindergarten meals + gender	Intercept	8.171	0.053	154.811	<0.001
	Kindergarten meals (CG)	0.031	0.068	0.454	0.652
	Gender (male)	-0.066	0.067	-0.989	0.328
Adjusted *R*-squared −0.021, *F*-statistic 0.5166 on 2 and 46 DF, *p* value 0.6					
d-ROM ~ kindergarten meals + gender	Intercept	340.604	15.652	21.762	<0.001
	Kindergarten meals (CG)	32.881	20.236	1.625	0.111
	Gender (male)	8.981	19.769	0.454	0.652
Adjusted *R*-squared 0.028, *F*-statistic 1.679 on 2 and 46 DF, *p* value 0.198

### Median levels of six urinary OSBs in healthy 5–6-year-old children

Median levels on creatinine-adjusted MDA, 8-OHdG, 8,15-PGF2α, 8-PGF2α, 11-PGF2α, and 15-PGF2 in fasting urine samples were determined for all 57 healthy 5–6-year-old children before intervention started (on day 1). All six OSBs were detected, but at varying frequencies, as shown in Table 4 alongside the IQR, minimum, and maximum values.

**Table 4:** Median levels of six urinary oxidative stress biomarkers in healthy 5–6-year-old children

	8-OHdG ng/mg urine creatinine	MDA ng/mg urine creatinine	8,15-PGF_2α_ ng/mg urine creatinine	8-PGF_2α_ ng/mg urine creatinine	11-PGF_2α_ ng/mg urine creatinine	15-PGF_2α_ ng/mg urine creatinine
Median (IQR) (min–max)	7.78 (4.75) (3.56**–**23.12)	9.59 (7.46) (1.01**–**91.57)	2.26 (3.94) (0.04**–**29.43)	0.21 (0.15) (0.08**–**2.35)	2.66 (1.13) (0.91**–**4.00)	1.42 (1.03) (0.62**–**3.33)
Detection frequencies *n*, (%)	57 (100)	57 (100)	34 (60)	14 (25)	4 (7)	12 (21)

### Urinary OSB levels in relation to the intervention trial

The intra-individual changes in MDA, 8-OHdG, 8,15-PGF2α, 8-PGF2α, 11-PGF2α, and 15-PGF2 concentrations in fasting urine measured on days 1 and 15 of the intervention kindergarten meals were examined. Levels of all OSB concentrations are shown in Supplementary Table S7. Due to low detection rate of 8-PGF2α, 11-PGF2α, and 15-PGF2α in our samples, a statistical analysis for changes in the concentration of these OSBs was not performed. A significant decrease in 8,15-PGF2α was detected in the urine of PG participants (*p* = 0.030), while changes in MDA and 8-OHdG were not significant (*p* = 0.765 and 0.536). No significant changes were found in the urinary OSBs in CG participants (*p* = 0.125, 0.487, and 0.263, respectively).

## Discussion

To the best of our knowledge, this is the first study to investigate the effects of antioxidant-rich kindergarten meals on oxidative stress biomarkers in the serum and urine of 5–6-year-old children. As demonstrated in our previous studies, kindergartens in PG offered meals with a significantly higher value of dTAC compared to kindergartens in CG [33]. Additionally, PG participants, on average, consumed significantly more antioxidants-rich foods due to a well-designed and precisely executed kindergarten meal plan [26]. All previous results are consistent with the results in the current study, reflecting a significantly higher average dTAC intake among PG participants, namely, at values that could have a health-protective effect. This is supported by two recent meta-analyses, which reported that the risk of all-cause mortality decreased significantly and linearly along with the 5.000 µmol/day increase in dTAC [12, 13]. Antioxidant-rich food also showed a moderate effect on obesity-induced low-grade inflammation in the HELEN study among teenagers from 10 European countries [16].

Furthermore, compared to the obese high-responder children in a 10-week intervention program [15], PG participants have a similar weekly average dTAC intake (6.300 vs. 6.200 µmol/day) indicating that a kindergarten diet rich in antioxidants could have a protective role against overweight and obesity. In addition, dTAC values around 10.000 µmol/day are associated with improved lung function among children with asthma [18] and a decreased risk of developing sensitization to inhalant allergens between 8 and 16 years of age [17]. dTAC values exceeding 8.000 µmol/day are positively associated with telomere length in the children and adolescent population [14].

The benefits of an antioxidant-rich kindergarten diet in our study were also reflected in a weak but significant negative correlation between dTAC intake and serum OSBs in our participants and in a significant decrease in intra-individual level of urinary 8,15-PGF2α in PG participants. However, the other OSBs did not show significant changes in either PG or CG participants. The most likely cause of the lack of differences is our short intervention period. It has been previously proposed that long-term interventions (> 8 weeks) seem to be more effective than short-term interventions [34]. However, the reduction of 8,15-PGF2α can be explained in the context of a review article discussing nutritional interventions in connection with the modulation of isoprostanes. The review showed that there was a reduction in F2-isoprostanes even in some short-term studies [35].

Our study was limited to a single blood sample, so we cannot provide an assessment of the changes in serum OSBs between days 1 and 15 of the intervention. However, the original data on d-ROM, PAT, and OSI levels in healthy 5–6-year-old Caucasian children may help fill the existing gap in lack of a universally accepted interval of normality [36]. d-ROMs in our participants ranged in value that was similar to d-ROMs in healthy 2–6-year-old Japanese children [9] but higher than d-ROMs in healthy 4-year-old Macedonian children [37]. Morimoto et al. report that d-ROMs decrease with increasing age and stabilize at 10–12 years [9].

MDA, 8-OHdG, and F2 isoprostanes are among the most widespread biomarkers of oxidative stress [7], but there is limited information on reference values in healthy children, despite the great interest due to the association of oxidative stress with NCDs and nutrition [5, 12]. To the best of our knowledge, this is the first study showing the concentrations of six urinary OSBs (MDA, 8-OHdG, 11-PGF_2α_ 8-PGF_2α_, 15-PGF_2α_, and 8,15-PGF_2α_) in healthy 5–6-year-old children. To avoid any bias due to the kindergarten diet, as average values, we present the concentrations of OSBs in the fasting urine before the intervention trial started.

The strength of our research is (1) the originality of the data on daily dTAC intake in kindergarten and outside kindergarten; (2) data obtained from consecutive 7–day dietary records; (3) presentation of the original levels of simultaneously measured urinary 8-OHdG, MDA, and the four isoprostanes in healthy 5–6-year-old children; (4) providing insight into serum d-ROM, PAT, and OSI values in healthy 5–6-year-old children; and (5) highlighting the positive impact of an antioxidant-rich kindergarten diet provides a strong rationale for further clinical and epidemiological studies in the context of health and early childhood nutrition.

This study has several limitations due to the short-term intervention design and the relatively small number of participants. Therefore, further long-term studies with follow-up and a larger number of participants are needed to confirm the established connection between antioxidant-rich kindergarten meals and changes in urinary and serum OSBs.

In conclusion, the results of our study highlight that antioxidant-rich kindergarten nutrition significantly increased the intake of dTAC in 5–6-year-old children, to a value that could have a protective health effect. This was further confirmed by the negative correlation between dTAC intake and serum OSBs (d-ROMs and OSI) in 5–6-year-old children as well as a significant decrease in 8,15-PGF2α in PG participants. Given the increasing number of children enrolled in kindergartens and the high burden of NCDs also among children, our findings underscore the need to promote antioxidant-rich food in kindergartens to promoting long-term health.

### Supplementary Information

Below is the link to the electronic supplementary material.

Supplementary file1 (DOCX 46 KB)

## Data Availability

The datasets used during the current study are available from the corresponding author on reasonable request.

## Abbreviations

CG: Control group
CVD: Cardiovascular diseases
dTAC: Dietary total antioxidant capacity
d-ROMs: Reactive oxygen metabolites
MDA: Malondialdehyde
NCDs: Noncommunicable diseases
ORAC: Oxidative radical absorbance capacity
OS: Oxidative stress
OSBs: Oxidative stress biomarkers
OSI: Oxidative stress index
PAT: Total antioxidant power
PG: Prototype group
8-OH-dG: 8-Hydroxy-2-deoxyguanosine
8-PGF2α: 8-Iso-prostaglandinF2α
11-PGF2α: 11β-ProstaglandinF2α
15-PGF2α: 15(*R*)-Prostaglandin F2α
8,15-PGF2α: 8-Iso,15(*R*)-prostaglandinF2α
