# Supplementary material for: Effect of antioxidant-rich kindergarten meals on oxidative stress biomarkers in healthy 5–6-year-old children: a randomized controlled trial
Source: Eur J Pediatr. 2024 Apr 25;183(7):3085–94. doi: 10.1007/s00431-024-05576-6 (PMC11192674; doi:10.1007/s00431-024-05576-6)
Supplement: Supplementary file 1 — Supplementary file1 (DOCX 46 KB) [file 431_2024_5576_MOESM1_ESM.docx]

**Effect of antioxidant-rich kindergarten meals on oxidative stress biomarkers in healthy 5–6-year-old children: a randomized controlled trial**

Maja Berlic^1,2^, Mojca Korošec^1^, Žiga Iztok Remec^3^, Vanja Čuk^3^, Tadej Battelino^4,5^, Barbka Repič Lampret^3^

**Affiliations**: ^1^Department of Food Science and Technology, Biotechnical faculty, University of Ljubljana, Ljubljana, Slovenia, ^2^Preschool Galjevica, Ljubljana, Slovenia, ^3^Clinical Institute for Special Laboratory Diagnostics, University Children’s Hospital, University Medical Centre Ljubljana, Ljubljana, Slovenia, ^4^Department of Endocrinology, Diabetes and Metabolic Diseases, University Children’s Hospital, University Medical Centre Ljubljana, Ljubljana, Slovenia, ^5^Faculty of Medicine, University of Ljubljana, Ljubljana, Slovenia

**Corresponding author:** Barbka Repič Lampret, [**barbka.repic@kclj.si**](mailto:barbka.repic@kclj.si)

**Supplementary material**

**Table S1.** Prototype 10-day kindergarten menu, including the recommended serving portions (g/child) and approximate energy intakes by individual meals ……………………………………………………………….…2

**Table S2.** Standard 10-day kindergarten menu from suburban area including the recommended serving portions (g/child) and approximate energy intakes by individual meals ………………………………………………..…..4

**Table S3.** Standard 10-day kindergarten menu from urban area including the recommended serving portions (g/child) and approximate energy intakes by individual meals…………………………………………………….6

**Methods for determining serum OSBs**…………………………………………………………………………..8

**Method for determining urinary OSBs**…………………………………………………………………………..8

**Table S4.** UHPLC flow profile and mobile phases ratio……………………………………………...………..….9

**Table S5.** Retention Time and Compound Dependent Mass Spectrometric Parameters…………...…………..…9

**Table S6.** dTAC intake according to eating location……………………………………………………...……...10

**Table S7.** Intra-individual concentrations (µmol/mol creatinine) of six oxidative stress markers in urine samples collected on day 1 and on day 15 of intervention kindergarten meals, from healthy 5–6-year-old children, randomized to prototype group (PG, n=40) and control group (CG, n=17); and detection frequencies for each OSBs………………………………………………………………………………………………………………11

**Table S1.** Prototype 10-day kindergarten menu, including the recommended serving portions (g/child) and approximate energy intakes by individual meals

|  | **Breakfast 20%^a^** | **Morning snack 5%^a^** | **Lunch 30-35%^a^** | **Afternoon snack 15%^a^** |
| --- | --- | --- | --- | --- |
| 1^st^ DAY Thursday | peppermint tea with honey (150g), whole grain bread (60g), tuna in olive oil(40g), turnips(50 g); apple(20 g) | blueberries (50g), raspberries  (50g) | bean soup with pasta(150g), vegetable patty(50g), mashed potatoes(120g), spinach(50g), beetroot salad (59g) | fruit-vegetable smoothie(130g), oatmeal bun(40g) |
| 2^nd^ DAY  Friday | cereal coffee with milk (160 g), scrambled eggs (60g), soy bread(60g), red pepper(50g); apple(20g) | strawberries (100g) | cauliflower soup(150g), buckwheat porridge  with asparagus(100g), veal steak(50g) in  natural sauces(90g), green salad(50g) | energy nut bar(30g), lemonade(150g) |
| 3^rd^ DAY  Monday | oatmeal with milk  (200g), ground walnuts  and cinnamon(20g), apple (20g) | pomegranate  (50g), kiwi  (50 g) | broccoli soup(150g), whole grain meat-vegetable lasagna(150g), green salat with corn (50g) | orange(100g), corn bombette(40g) |
| 4^th^ DAY Tuesday | hot cocoa(150 g) three grains bread(60g), butter (20g), raspberry jam(10g); apple(20 g) | green pepper (20g), yellow pepper(10g), red pepper (20g) | tomato soup(150g), chicken fillet with sesame(60g), baked potatoes(120g), beetroot salad(59 g) | buckwheat bread (40g), cheese(20g), walnuts(5g), black olives(10g), cherry tomatoes(30g) |
| 5^th^ DAY Wednesday | herbal tea with honey and lemon(150g), bread with seeds(60g), spinach spread(30g), cherry tomato(50g) | blueberries (100g) | lentil soup(150g), baked salmon(60g) roasted brussels sprouts(20g), wholemeal polenta(120g) with parmesan cheese(3g), radish salad(50g) | cottage cheese(80g) with strawberry porridge(30g), sesame bread(40g) |
| 6^th^ DAY Thursday | hot cocoa(180g),  wheat germ bread(60g), cottage spread with chives (30g), apple(20g) | cherry tomatoes (50g), radish (50g) | beef soup(170g) with noodles(50g), beef(60g), sauté potatoes(130g), steamed broccoli(25g) and cauliflower(25g), red cabbage salad(50g) | blueberries(100g), buckwheat bread with walnuts(40g) |
| 7^th^ DAY  Friday | herbal tea with honey and lemon(150g), spelt bread (70g), egg spread with vegetable and chicken breast salami(30g), orange pepper(50g), apple(20g) | kiwi(50g), raspberry (50g) | kale stew with chicken and quinoa(260 g), rye bread(30 g), yogurt panna cotta(80 g) with blueberry sauce(50 g), 100% orange juice(120 g) | spelt bread roll (50g), red pepper (50g), black olives (6g), cheese(20g) |
| 8^th^ DAY  Monday | millet porridge with milk (280g), cinnamon and ground chocolate topping (3g), apple(20g) | dried cranberries (15g) and plums(25g), walnuts(15g) | broccoli soup(188g), whole grain spaghetti(130g), tuna in tomato sauce with pine nuts and basil(147g), green salad(50g) with pomegranate(5g) | apple sauce with cinnamon and cloves(130g), whole  grain breadsticks (grissini)(20g) |
| 9^th^ DAY Tuesday | peppermint tea with honey(150g), sesame bread roll (70g), chicken breast salami(15g), edam cheese(15g), tomato(50g), apple(20g) | strawberries  (100g) | barley stew with turkey (260g), rye bread(20g), buckwheat cubes with dark chocolate and apples(80g), lemonade(140g) | yogurt 100g) with raspberry puree(50g) and ground hazelnuts(20g),  multigrain bread(20g) |
| 10^th^ DAY Wednesday | cereal coffee with milk(160g), whole grain bread roll(60g), dried cranberries(30g), walnuts(30g), apple(20g) | blueberries (100g) | tomato soup(199g) with noodles(49g), baked brown rice with chicken and vegetables(203g), parmesan cheese(5g), beetroot salad(59g) | nuts bar(42g), 100% pomegranate juice(150g) |

^a^Distribution of recommended energy intake throughout the day by individual meals

**Table S2.** Standard 10-day kindergarten menu from suburban area including the recommended serving portions (g/child) and approximate energy intakes by individual meals

|  | **Breakfast 20%^a^** | **Morning snack 5%^a^** | **Lunch 30-35%^a^** | **Afternoon snack 15%^a^** |
| --- | --- | --- | --- | --- |
| 1^st^ DAY Thursday | buckwheat bread(85g), edamame cheese(25g), tomato(20g) | banana(30g),  apricot(5g) | vegetable risotto with barley and chicken(150g) green salad(20g) | grapes(80g), toast(50g) |
| 2^nd^ DAY  Friday | cereal coffee with milk (150g), cacao bun(40g) | banana(30g) | broccoli soup with fried balls(150g), torn pancakes(70g) | pear(100g), whole grain bread(85g) |
| 3^rd^ DAY  Monday | poppy seed bread(40g), milk(100g) | pear(150g) | macaroni(120 g), Bolognese sauce(60g), parmesan cheese(2g), cabbage salad(20g) | banana(200g), semi white bread(85g) |
| 4^th^ DAY Tuesday | black bread(80g), chicken breast(20g), red pepper(20g) | banana(50g),  melon(10g),  pear(5g) | potatoes and bean stew(130g) hot dog(40g), semi white bread(40g), pudding(100g) | baked bun with marmalade(60g), tea(120g) |
| 5^th^ DAY Wednesday | milk rice (250g), chocolate sprinkles(3g)/black bread(30g) | pear(40g),  apple(40g) | roast pork(50g), »mlinci«(60g), green salad(30g) | apple(50g), whole grain cookies(55g) |
| 6^th^ DAY Thursday | fruit tea with sugar(190 g), apricot jam croissant(85g) | banana(50g), pear(45g) | salmon fillet(70g), with sauce of garlic, parsley and olive oil(3g), boiled potatoes(200g), beetroot(90 g) | banana(130g), buckwheat bread(70g) |
|  |  |  |  |  |
| 7^th^ DAY  Friday | fruit tea(120 g), eco white bread(30 g), eco veal hot dog(50 g), mustard(10 g) | banana(60 g), pear(96 g) | chicken ragout  (120 g), wide white noodles(200 g), green lettuce(20 g) | eco black bread(30 g), apple(120 g) |
| 8^th^ DAY  Monday | herbal tea(120 g), semi white bread(50 g), cooked prosciutto(30 g), pickle  (10 g) | apple(40 g), pear(60 g) | beef steak(50 g) in carrot sauce(110 g) roasted potatoes(290 g), green salad(40 g) | banana(140 g), whole grain bread(30 g) |
| 9^th^ DAY Tuesday | cocoa(120 g), cheese bread roll(80 g) | apple(50 g), pear(50 g) | bean stew with white pasta(360 g), marble cake(50 g) | melon(100 g), whole grain cookies(20 g) |
| 10^th^ DAY Wednesday | white wheat grits in milk(190 g), chocolate topping(1 g) | apple(50 g), pear(50 g), banana(60 g) | risotto (rice, barley, spelt) with turkey and vegetables(260 g), green salad with boiled egg(60 g) | pear(120 g), breadsticks(grissini)  (20 g) |

^a^Distribution of recommended energy intake throughout the day by individual meals

**Table S3.** Standard 10-day kindergarten menu from urban area including the recommended serving portions (g/child) and approximate energy intakes by individual meals

|  | **Breakfast 20%^a^** | **Morning snack 5%^a^** | **Lunch 30-35%^a^** | **Afternoon snack 15%^a^** |
| --- | --- | --- | --- | --- |
| 1^st^ DAY Thursday | rye bread(60g), herbal spread(30g), herbal tea(150g) | apple(50 g), banana(50 g) | vegetable minestrone with chicken(200g), hazelnut pastry with fruit(60g) | apple(100g) |
| 2^nd^ DAY  Friday | black bread(60g), egg spread(30g), fruit tea(150g) | apple(50 g), pear(50 g) | macaroni**(**120 g), Bolognese sauce(60g), beetroot salad(40g) | whole grain toast(40g), nectarine(80g) |
| 3^rd^ DAY  Monday | oatmeal bread(60g)  sour cream(30g),  herbal tea(150g) | banana(50 g), pear(45 g) | whole grain pasta with beef(150g)  green salad(40g) with corn(10g) | bio yogurt green apple(150g)  rice waffles(40g) |
| 4^th^ DAY Tuesday | granola with cranberries(40g), milk(120g) | apple(50 g), banana(50 g) | potato goulash(180g), plum dumplings(60g) | bun with seed(40g), 100% apple juice (100g) |
| 5^th^ DAY Wednesday | buckwheat bread with walnuts(60g), edamame cheese(25g), fresh cucumber(20g)  fruit tea(150g) | apple(50 g), pear(50 g) | chicken paprikash(100g),  bio bulgur(100g)  bio beetroot salad(40g) | apple(100g) |
| 6^th^ DAY Thursday | eco milk (120 g), black bread (70 g), honey and butter spread (20 g) | apple  (80 g) | beef and vegetable lasagna (280 g), green salad with chickpeas (35 g) | banana (120 g) |
| 7^th^ DAY  Friday | tea (180 g), eco spelt bread (65 g), turkey salami(30 g), sour red pepper (15 g) | nectarine  (80 g) | barley stew (280 g),  cottage cheese strudel (110 g) | eco spelt bread (30 g), apple (90 g) |
| 8^th^ DAY  Monday | white coffee (140 g), semi white bread (65 g), butter (15 g), apricot jam (20 g) | nectarine  (80 g) | roasted chicken legs (45g), mashed potatoes (200 g), green salad with carrots (30 g) | rice waffle (10 g), apple (80 g) |
| 9^th^ DAY Tuesday | millet porridge with milk (240 g), chocolate topping (3 g) | banana (70 g), eco carrot  (10 g) | beef goulash (140 g), steamed bread dumplings (60 g), green salad with corn (30 g) | pear (80 g), whole grain toast (10 g) |
| 10^th^ DAY Wednesday | tea (300 g) eco whole grain bread roll (70 g), vegetable spread (20 g) | apricot  (80 g) | carrot soup (140 g), fried hake (80 g), salad with cabbage and potatoes (150 g) | vanilla yogurt (155 g), eco whole grain bread (20 g) |

^a^Distribution of recommended energy intake throughout the day by individual meals

**Methods for determining serum OSBs**

The d-ROMs test is based on the Fenton reaction. The ROMs (primarily hydroperoxides) in serum can generate aloxyl and peroxyl radicals in the presence of iron released from serum proteins with the help of an acid buffer. Hydroperoxides are converted into radicals that oxidize N,N-diethyl-para-phenylenediamine and can be detected spectrophotometrically using an automatic analyzer. Results for d-ROMs are expressed in Carratelli units (U.Carr), where one U.Carr corresponds to 0.08 mg H_2_0_2_/dL [1]. According to the manufacturer, the reference values between 250 and 300 U.Carr have been suggested.

The PAT test is designed to measure the plasma antioxidant capacity and it is based on the ability of plasma AOs to reduce ferric ions (Fe^3+^) to ferrous ions (Fe^2+^). To negate the overestimation of the plasma antioxidant capacity due to the concentration of phosphates in the serum, the presence of zirconium salts is needed in reaction mixture. Values are expressed in Cornelli units (U.Cor), where 1 U.Cor corresponds to 1.4 μmol/L of ascorbic acid [2]. The reference values between 2200 and 2800 U.Cor have been suggested.

The OSI was developed to summarize the information derived from the d-ROM analysis and the PAT analysis. It is not a simple arithmetic formula but a complex logical-mathematical algorithm that can weigh the information in a single value. The deviation of both values from normality, whether the values are raised or lowered, raises the OSI. The higher the value of the OSI index, the greater the deviation of the redox balance from normality. According to the manufacturer, the value range for the OSI index is from 0 to 200, with normal adult reference values below 40.

**Method for determining urinary OSBs**

Waters Acuity I-class UHPLC coupled with Waters Xevo TQ-S micro tandem mass spectrometer (Waters Corporation, Milford, MA, USA) was used for quantification of target analytes. Chromatographic separation of target analytes was performed using InfinityLab Poroshell 120 SB-AQ 2,7 um (2.1 × 100 mm) column (Agilent, Santa Clara, CA, USA). The mobile phase consisted of 0.01% acetic acid in water as solvent A and 0.01% acetic acid in methanol as solvent B. UHPLC flow profile and mobile phases ratio are described in detail in Table S4. Additionally, retention time and compound-dependent mass spectrometric parameters are described in detail in Table S5. Standard solution preparation was performed as previously described by Martinez and Kannan [3]. For sample preparation, we adhered to the procedure outlined by the authors, with the exception that we conducted solid phase extraction (SPE) using Evolute Express ABN columns (Biotage Sweden AB, Uppsala, Sweden).

**Table S4.** UHPLC flow profile and mobile phases ratio

| **Time (min)** | **Flow (mL/min)** | **% A** | **% B** | **curve** |
| --- | --- | --- | --- | --- |
| 0 | 0.300 | 80 | 20 | initial |
| 0.10 | 0.300 | 80 | 20 | 6 |
| 0.65 | 0.300 | 55 | 45 | 6 |
| 5.10 | 0.300 | 45 | 55 | 1 |
| 5.60 | 0.300 | 0 | 100 | 6 |
| 8.00 | 0.300 | 0 | 100 | 6 |
| 8.50 | 0.300 | 80 | 20 | 3 |
| 10.00 | 0.300 | 80 | 20 | 1 |

**Table S5.** Retention Time and Compound Dependent Mass Spectrometric Parameters

| **compound** | **RT^a^**  **(min)** | **ionization**  **mode** | **MRM^b^ transition**  **(m/z)** | **capillary voltage**  **(kV)** | **cone voltage**  **(V)** | **colision voltage**  **(V)** |
| --- | --- | --- | --- | --- | --- | --- |
| 8-OHdG | 1.55 | + | 284.10 > 167.99 | 2.5 | 24 | 10 |
| 15N5-8OHdG | 1.55 | + | 289.10 > 172.98 | 2.5 | 33 | 10 |
| MDA-DNPH | 2.22 | + | 235.10 > 158.99 | 2.5 | 25 | 20 |
| D2-MDA-DNPH | 2.22 | + | 237.00 > 161.10 | 2.5 | 14 | 20 |
| 8,15-PGF2a | 3.63 | - | 353.18 > 193.20 | 2.5 | 31 | 25 |
| 8-PGF2a | 3.83 | - | 353.18 > 193.20 | 2.5 | 31 | 25 |
| D4-8-PGF2a | 3.83 | - | 357.24 > 197.12 | 2.5 | 7 | 25 |
| 11-PGF2a | 3.97 | - | 353.18 > 193.20 | 2.5 | 31 | 25 |
| 15-PGF2a | 4.49 | - | 353.18 > 193.20 | 2.5 | 31 | 25 |

^a^RT – retention time; ^b^MRM – multiple reaction monitoring

References

1. Trotti R, Carratelli M, Barbieri M (2002) Performance and clinical application of a new, fast method for the detection of hydroperoxides in serum. Panminerva Med 44:37-40
2. Serena B, Primiterra M, Catalani S, Finco A, Canestrari F, Cornelli U (2013) Performance evaluation of the innovative PAT test, comparison with the common BAP test and influence of interferences on the evaluation of the plasma antioxidant capacity. Clin Lab 59:1091-1097
3. Martinez MP, Kannan K (2018) Simultaneous analysis of seven biomarkers of oxidative damage to lipids, proteins, and DNA in urine. Environ Sci Technol 52:6647-6655. <https://doi.org/10.1021/acs.est.8b00883>

**Table S6.** dTAC intake according to eating location

|  | Weekday in kindergarten | Weekday outside kindergarten | Total  weekday | Total  weekend | 7-days average |
| --- | --- | --- | --- | --- | --- |
| PG (µmol TE)  Mean ±SD | 8.558±5.361 | 4.070±4.616 | 11.612±6816 | 5.550**±**4.371 | 6.287±5.317 |
| CG (µmol TE)  Mean ±SD | 2.831**±**1.957 | 3.391±3.338 | 5.499±4107 | 5.243±5.687 | 3.505±3.555 |
| p value | <0.001* | 0.196 | <0.001* | 0.779 | <0.001* |

PG – Prototype group; CG – Control group

**Table S7.** Intra-individual concentrations (µmol/mol creatinine) of six oxidative stress markers in urine samples collected on day 1 and on day 15 of intervention kindergarten meals, from healthy 5–6-year-old children, randomized to prototype group (PG, n=40) and control group (CG, n=17); and detection frequencies for each OSBs

|  |  | **8-OHdG**  (µmol/mol creatinine) | **MDA**  (µmol/mol creatinine) | **8,15-PGF_2α_** (µmol/mol creatinine) | **8-PGF_2α_** (µmol/mol creatinine) | **11-PGF_2α_** (µmol/mol creatinine) | **15-PGF_2α_** (µmol/mol creatinine) |
| --- | --- | --- | --- | --- | --- | --- | --- |
| PG 1^st^ day | Median(IQR) | 3.32(2.23) | 14.5(14.41) | 0.51(1.17) | 0.06(0.05) | 0.29 | 0.54(0.34) |
|  | Detection frequencies n(%) | 40(100) | 40(100) | 26(65) | 13(32) | 1(3) | 11(28) |
| PG 15^th^ day | Median(IQR) | 3.42(1.37) | 15.94(19.38) | 0.34(0.4) | 0.08(0.03) | 1.73(1.13) | 0.47(0.77) |
|  | Detection frequencies n(%) | 40(100) | 40(100) | 25(63) | 13(33) | 4(10) | 23(58) |
|  | ^a^Number of pairs | 40 | 40 | 17 | 1 | 0 | 4 |
| p-value | | 0.536 | 0.765 | 0.030* | ^b 2^/ | ^b 2^/ | ^b 2^/ |
| CG 1^st^ day | Median (IQR) | 2.76(0.6) | 16.05(7.48) | 1.4(1.26) | 0.1 | 0.93(0.25) | 0.3 |
|  | Detection frequencies n(%) | 17(100) | 17(100) | 8(47) | 1(6) | 3(18) | 1(6) |
| CG 15^th^ day | Median (IQR) | 2.71(1.3) | 16.21(10.97) | 0.21(0.14) | 0.13(0.12) | 1.47(0.4) | 0.8(0.63) |
|  | Detection frequencies n(%) | 17(100) | 17(100) | 13(76) | 7(41) | 4(24) | 7(41) |
|  | ^a^Number of pairs | 17 | 17 | 5 | 1 | 1 | 0 |
| p-value | | 0.263 | 0.487 | 0.125 | ^b 2^/ | ^b 2^/ | ^b 2^/ |

PG – Prototype group; CG – Control group

^a^number of detected OSBs in both urines in individuals

^b^due to the insufficient number of detected OSBs among individual pairs, we did not perform a statistical analysis
